# Interpreting Abdominal Surgical-Site Infection Trends Across the COVID-19 Shock: A Critical Narrative Review and the SSI Signal-Validity Framework

**DOI:** 10.3390/life16081320

**Published:** 2026-08-12

**Authors:** Emil-Tiberius Trasca, Dumitru Radulescu, Eleonora Daniela Ciupeanu-Calugaru, Razvan Mercut, Vlad Dumitru Baleanu, Elena-Irina Caluianu, Alexandru-Marian Vieru, Patricia-Mihaela Radulescu

**Affiliations:** 1 Department of Surgery, University of Medicine and Pharmacy of Craiova, 200349 Craiova, Romania; etrasca@yahoo.com (E.-T.T.); dr_radulescu_dumitru@yahoo.com (D.R.); rui_costa23@yahoo.com (E.-I.C.); 2 Department of Biology and Environmental Engineering, University of Craiova, 200585 Craiova, Romania; 3 Department of Plastic and Reconstructive Surgery, University of Medicine and Pharmacy of Craiova, 200349 Craiova, Romania; razvan.mercut@umfcv.ro; 4 Department of General Surgery, St. Pantelimon Emergency Clinical Hospital, 021659 Bucharest, Romania; baleanuvlad@gmail.com; 5 Doctoral School, University of Medicine and Pharmacy of Craiova, 200349 Craiova, Romania; alexandruvieru1993@yahoo.com; 6 Department of Pulmonology, University of Medicine and Pharmacy of Craiova, 200349 Craiova, Romania; paty_miha@yahoo.com

**Keywords:** surgical site infection, abdominal surgery, COVID-19, case mix, post-discharge surveillance, ascertainment bias, infection depth, health-system resilience

## Abstract

**Background/Objectives:** Comparisons of abdominal Surgical-Site Infection (SSI) rates during COVID-19 often treat recorded incidence as a direct measure of biological risk, although the pandemic altered operative volume and case mix, prevention practices, and post-discharge detection. This review examines when reported SSI changes are interpretable. **Methods:** We conducted a critical narrative review, informed by SANRA and critical interpretive synthesis, of MEDLINE/PubMed, Embase, Scopus, and Europe PMC (January 2018–25 June 2026). Comparative SSI studies formed the core evidence set, supported by contextual studies on emergency presentation, biological risk, prevention, and surveillance. **Results:** Studies reported lower, unchanged, non-monotonic, and higher SSI rates. These differences became more coherent when the recorded rate was separated into operative composition, true infection probability, and detection probability. No core study measured all domains required to isolate a biological pandemic effect. Evidence from acute pancreatitis and abdominal trauma showed that disease severity, care access, inflammatory status, and prognostic relationships varied with health-system context. **Conclusions:** We propose the Surgical-Site Infection Signal-Validity Framework (SSI-SVF). It classifies findings as probable true improvement, apparent improvement, masked deterioration, mixed deterioration, or indeterminate and requires joint assessment of operative composition, true infection risk, and detection probability before interpreting a calendar-period change as altered surgical safety.

## 1. Introduction

### 1.1. The Question Is Not Merely Whether SSI Increased or Decreased

Surgical-Site Infection is a major source of postoperative morbidity after abdominal surgery, but its reported incidence is inseparable from the surveillance system used to find it. Standard frameworks define procedure-specific surveillance windows; distinguish superficial incisional, deep incisional, and organ/space infection; and expect active case finding after discharge [1,2,3,4]. In a prospective international cohort of gastrointestinal surgery, the burden of SSI varied markedly across settings and was strongly related to procedure and patient risk [5]. Thus, a recorded SSI rate is not a direct readout of microbial biology; it is the endpoint of a clinical and measurement pathway.

The COVID-19 pandemic disturbed every component of that pathway. Elective operations were cancelled or postponed at unprecedented scale, while cancer and urgent surgery were preferentially preserved [6]. Patients who did undergo elective surgery were therefore selected from a different risk pool. Emergency presentations were frequently delayed, increasing contamination and physiological derangement. At the same time, hospitals intensified some infection-control practices but experienced staffing shortages, supply constraints, and disruption of routine prevention work [7,8,9,10,11,12]. Finally, early discharge, reduced outpatient contact, and remote review altered the probability that a postoperative wound infection would be identified.

Published comparisons consequently point in every direction. Some studies report lower SSI during the first pandemic year [13,14,15,16,17,18], some report no material difference [19,20], and others report higher or rebounding rates [15,21,22,23]. These differences are often framed as a simple contest between better prevention and greater harm. A more fundamental problem is that the studies often estimate different quantities with different denominators and different detection sensitivity. A change in the recorded rate can therefore occur without a corresponding change in true risk, while a clinically important deterioration can be hidden inside an unchanged aggregate rate.

### 1.2. Objective and Original Contribution

We kept a narrative design because the task here is to explain and interpret, not to estimate a single pooled pandemic effect. Its objective is to determine when a reported pre-pandemic, pandemic, or post-pandemic SSI difference can be interpreted as a change in true surgical safety and when it is more likely to reflect denominator selection, depth-specific masking, prevention-process failure, or incomplete surveillance. We call the resulting construct the Surgical-Site Infection Signal-Validity Framework (SSI-SVF). Its canonical proposition is that a calendar-period change in recorded SSI is interpretable as a change in surgical safety only when operative composition (w), true stratum-specific infection probability (p), and detection probability (q) are considered jointly.

The review makes four linked contributions:

- It separates population burden, recorded per-procedure risk, and true per-procedure risk—three outcomes that can move in opposite directions;
- It replaces an additive “pandemic effect” with a measurement-aware decomposition in which procedure/case-mix weights, true infection probability, and detection probability are distinct;
- It audits the interpretability of core comparative studies across seven explicitly defined domains, including post-discharge completeness and prevention-process measurement;
- It proposes the SSI-SVF five-class signal-validity matrix and a minimum reporting set that generate testable rules for future system shocks and structured evidence synthesis.

The aim is therefore not to restate that elective surgery fell while emergency disease grew more severe. It is to give clinicians, surveillance teams, and researchers a shared way of judging what a given SSI signal can and cannot mean, and of saying plainly where the evidence runs out.

## 2. Materials and Methods

### 2.1. Review Design

We conducted a critical narrative review informed by the Scale for the Assessment of Narrative Review Articles (SANRA) and by principles of critical interpretive synthesis [24,25]. SANRA was used to strengthen justification, search transparency, scientific reasoning, and presentation; it was not treated as a risk-of-bias instrument. Critical interpretive synthesis was used as a methodological orientation because it permits iterative movement between empirical findings and an emerging explanatory construct when conventional effect pooling would obscure the problem under study.

The review was deliberately organized around a causal-measurement question: “What combination of case selection, true risk, and surveillance can produce the reported direction of change?” It does not claim exhaustive systematic coverage, a pooled effect estimate, or causal identification of the pandemic. We did not follow the PRISMA reporting guideline, which is intended for systematic reviews and meta-analyses; instead, the search and charting steps were documented transparently so that the reasoning can be inspected and reproduced rather than to claim systematic-review status. We also considered the PRISMA extension for scoping reviews (PRISMA-ScR). We did not adopt it as a formal reporting standard, because the sampling was purposive rather than exhaustive and because the intended output is an interpretive framework rather than a map of a field. We have, however, followed its reporting logic for the elements that determine reproducibility: databases, date limits, eligibility for the core comparative set, and the number of records retained at each stage are stated in Section 2.2, and the charting form is stated in Section 2.3. The evidence base should therefore be read as transparently documented but not exhaustive, and the selection as purposive rather than protocol-driven.

### 2.2. Information Sources and Search Strategy

MEDLINE/PubMed, Embase, Scopus, and Europe PMC were searched from January 2018 to 25 June 2026. Search terms combined “surgical site infection” or “surgical wound infection” with abdominal, general, colorectal, colectomy, cholecystectomy, laparotomy, or emergency-surgery terms and COVID-19, SARS-CoV-2, pandemic, lockdown, or post-pandemic terms. A supplementary contextual search combined SSI or postoperative infection with post-discharge surveillance, electronic health records, and automated case finding. Disease-specific abdominal-emergency studies, including acute pancreatitis and abdominal trauma, were identified through targeted citation chasing when they informed case-mix, host-response, or transportability mechanisms. Reference lists of eligible reports, recent reviews, and surveillance guidance were searched iteratively. English- and Romanian-language full texts were considered. The 2018 start date provided a recent pre-pandemic baseline while allowing inclusion of studies that used 2018–2019 comparators.

**Figure 1:**
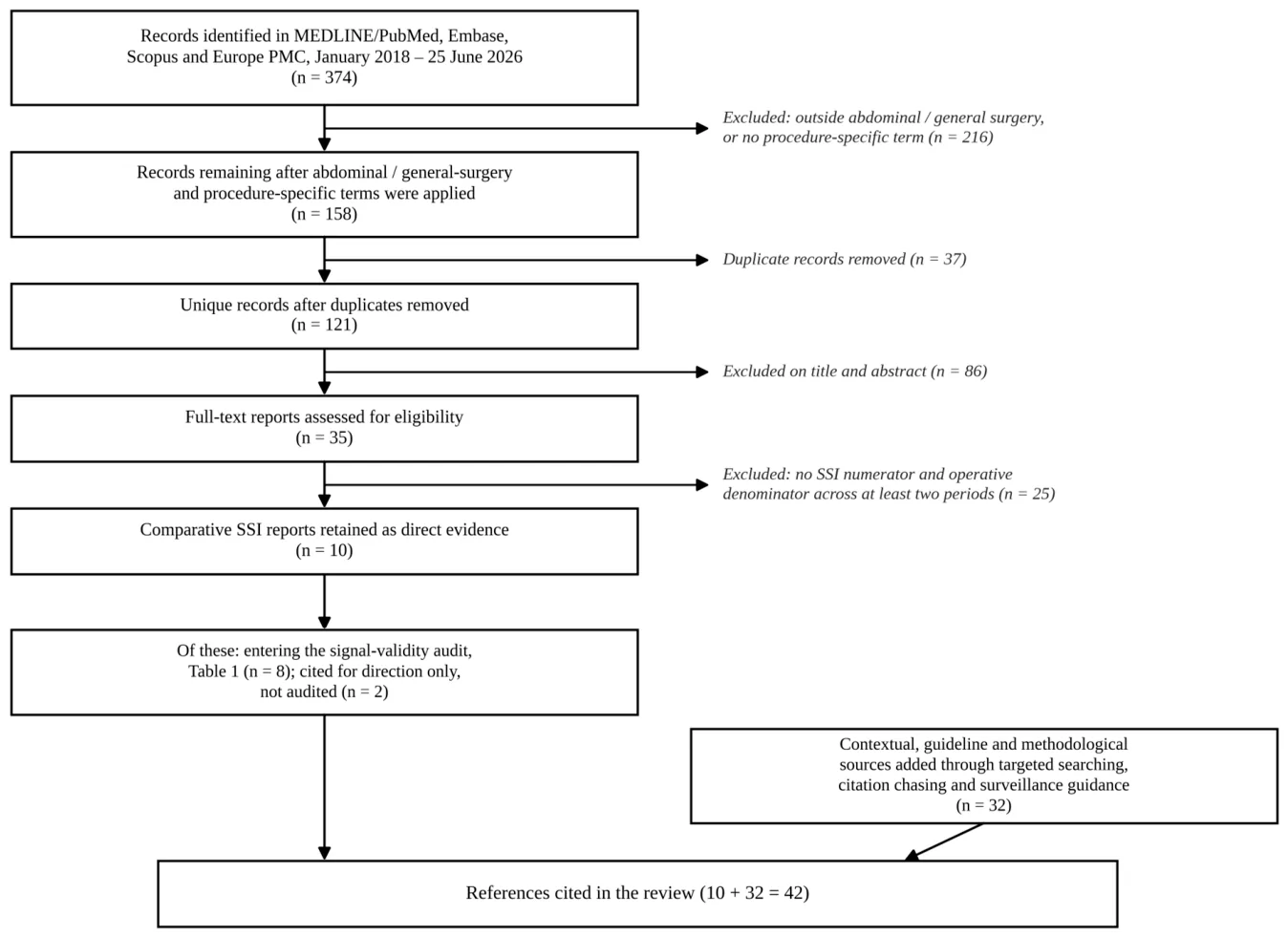
Flow of records through the search and selection process. The counts are those reported in Section 2.2. The review is a critical narrative review, and this diagram is given for transparency of selection; it does not indicate a systematic or scoping-review protocol.

Sampling had two evidence roles. The core comparative set comprised studies that reported an SSI numerator and operative denominator across at least two calendar phases, or used an interrupted time-series design, for adult abdominal/general-surgery populations or an extractable relevant subgroup. A contextual set included studies of emergency presentation, acute pancreatitis, abdominal trauma, procedure-specific biological heterogeneity, healthcare-associated infection, prevention-process disruption, post-discharge surveillance, and electronic-record case finding. Contextual studies were not counted as direct evidence that SSI changed; they were used to evaluate the plausibility of mechanisms and the requirements for stable outcome measurement. The database searches yielded 374 records. Applying the abdominal/general-surgery and procedure-specific terms reduced these to 158, and 121 unique records remained after deduplication, of which 35 full texts were assessed. Ten comparative SSI reports were retained as direct evidence; eight of them met the stricter criteria for the formal signal-validity audit reported in Table 1 [13,14,15,16,19,20,21,22], while two were cited for direction only and not audited [17,18]. Targeted contextual searching, citation chasing, and surveillance guidance contributed a further 32 sources, giving the 42 references cited in this review. The flow of records through these stages is shown in Figure 1.

### 2.3. Study Charting and the Signal-Validity Audit

For each core comparative study, the review team charted setting, procedure family, urgency, period definition, denominator, SSI definition and surveillance window, method and completeness of post-discharge ascertainment, depth-specific outcomes, case-mix variables, prevention-process indicators, and analytic time structure. We then classified reporting in seven signal-validity domains: (1) standardized definition and fixed window; (2) explicit post-discharge method and completeness; (3) procedure and urgency stratification; (4) SSI depth stratification; (5) case-mix measurement or adjustment; (6) direct measurement of prevention-process fidelity; and (7) a time structure capable of distinguishing abrupt change from pre-existing or rebound trends.

The audit was not converted into a numerical quality score. Missing information in one domain does not make a study intrinsically poor; it limits which causal interpretation is defensible. For example, a well-adjusted cohort can estimate an association between calendar period and recorded SSI while remaining unable to distinguish a true reduction from reduced post-discharge detection if surveillance completeness is unreported.

### 2.4. Synthesis

**Figure 2:**
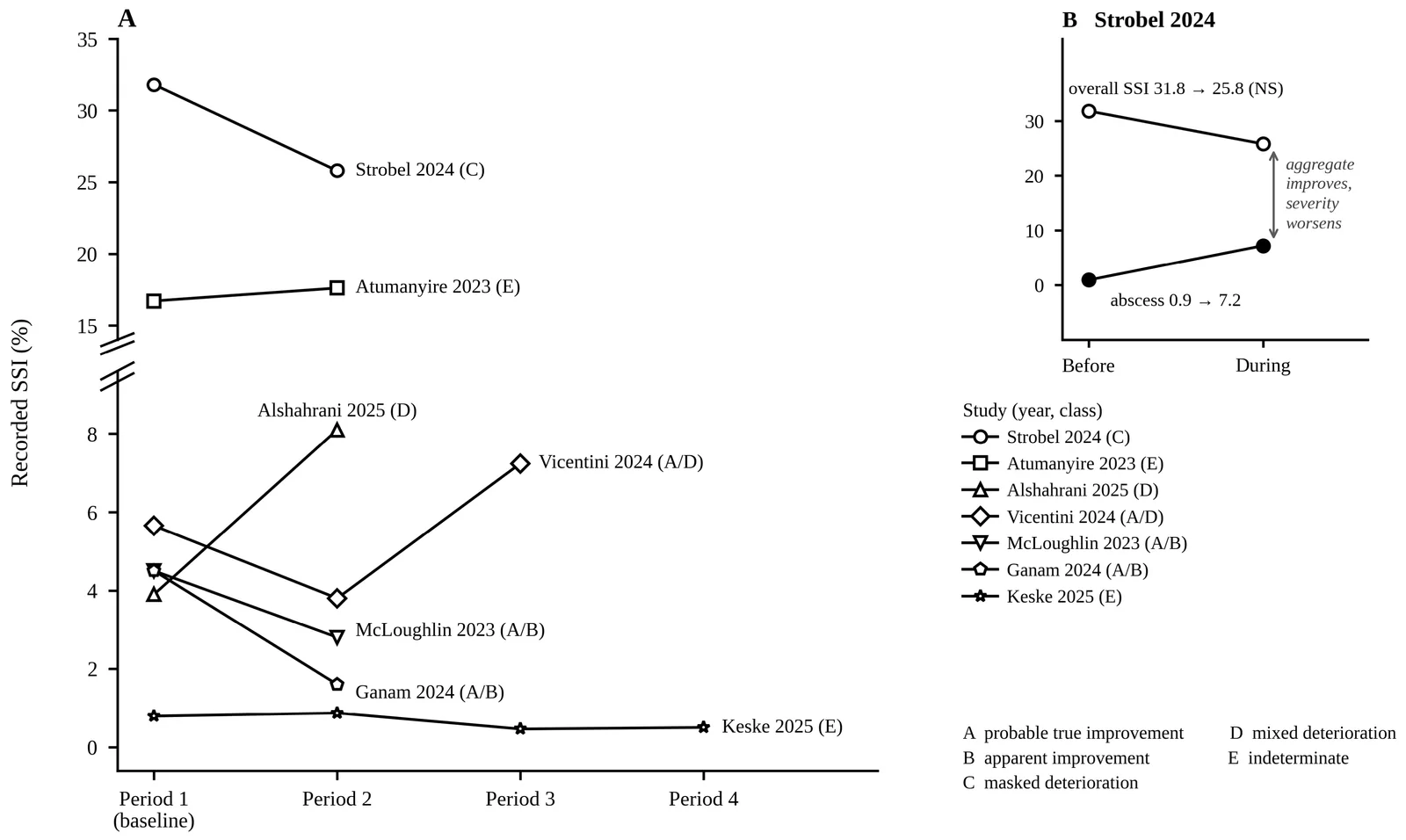
Recorded SSI trajectories across core comparative studies, coloured by the signal-validity class assigned in Table 1. Panel (**A**) shows the recorded SSI signal reported in each study across its sequential calendar periods; Panel (**B**) isolates Strobel et al. [19] to show how a flat or improved aggregate can coexist with a rising severity-specific outcome. Values are the recorded percentages reported in the cited sources, transcribed without smoothing, pooling, or estimation. Panel (**A**) uses a broken vertical axis so that the two high-incidence cohorts and the remaining studies are both legible; study identity is carried by the marker symbol and the direct label rather than by colour. Smith et al. [14] reported a non-significant colorectal-subgroup decrease without a stated point estimate and is therefore summarized in Table 1 only. Marker shape identifies the study—circle, Strobel et al. [19]; square, Atumanyire et al. [20]; upward triangle, Alshahrani et al. [22]; diamond, Vicentini et al. [15]; downward triangle, McLoughlin et al. [16]; pentagon, Ganam et al. [13]; star, Keske et al. [21]—and the key on the right repeats each symbol with its reference number and Table 1 class. In Panel (**B**) the open circles are the overall SSI rate and the filled circles the intra-abdominal abscess rate.

**Figure 3:**
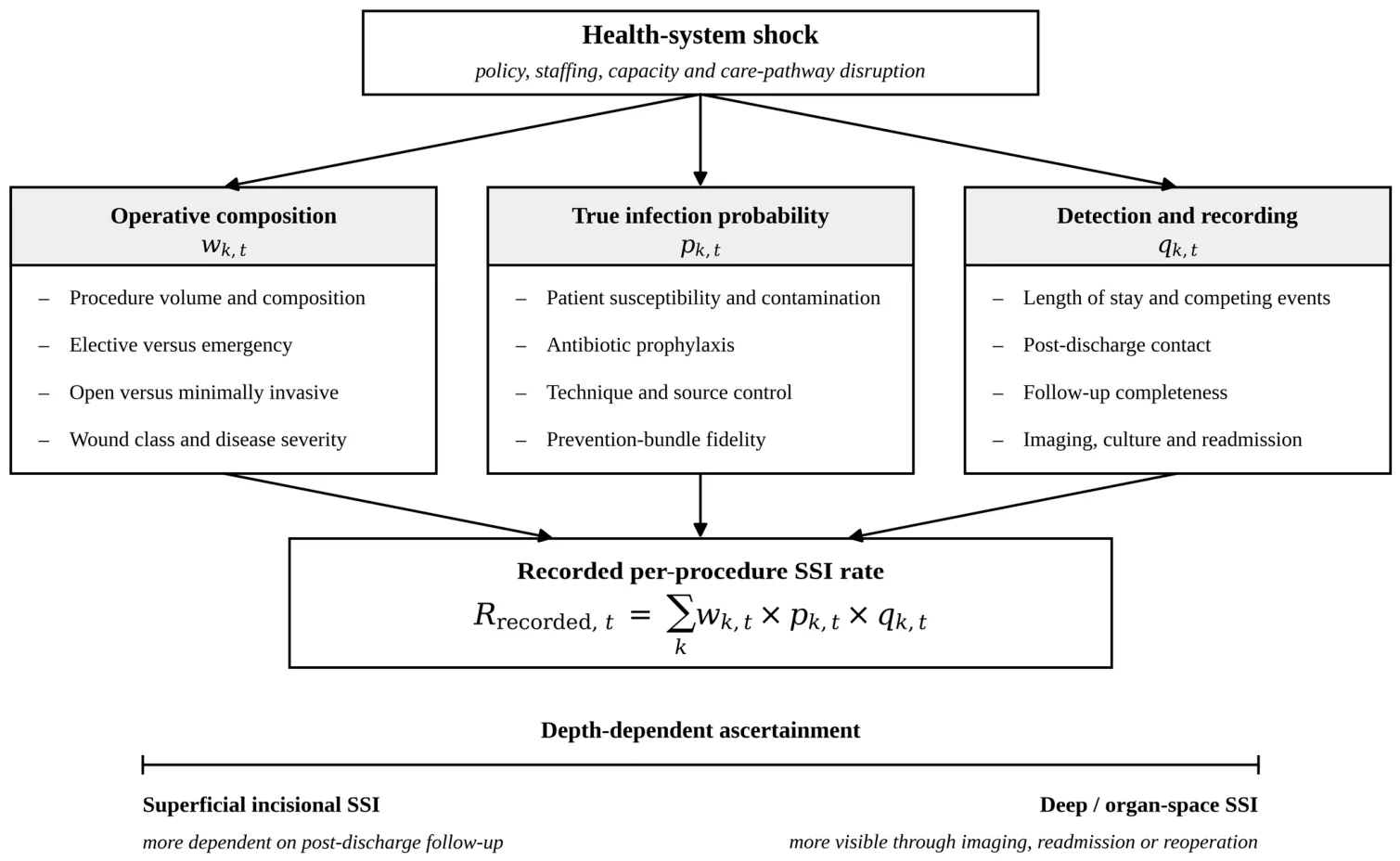
Measurement-aware decomposition of the recorded SSI rate. Operative composition (w), true stratum-specific infection probability (p), and detection probability (q) can all change during a system shock; surveillance dependence also varies by SSI depth.

**Figure 4:**
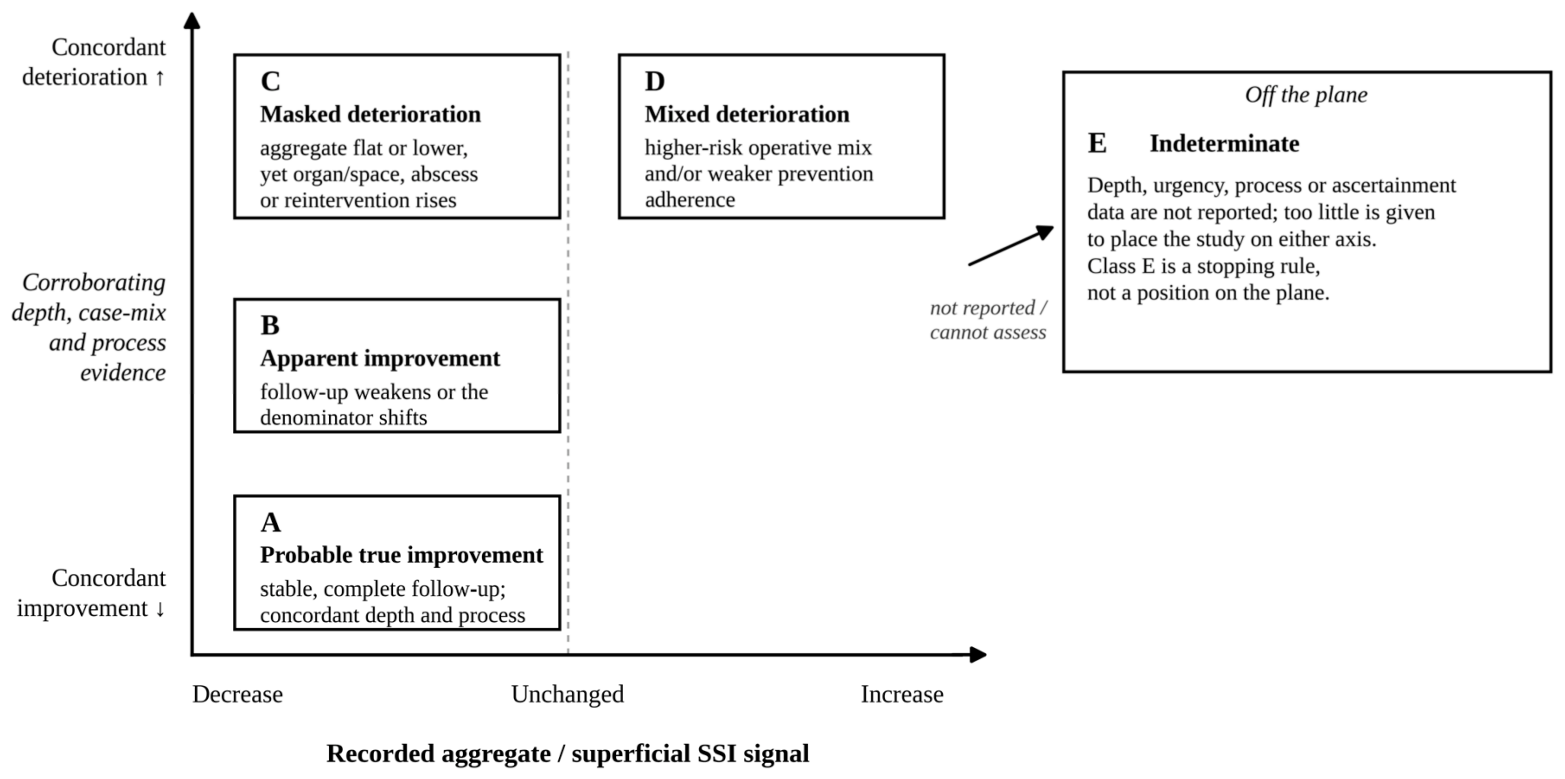
SSI-SVF signal-validity matrix. The horizontal axis is the recorded aggregate/superficial SSI direction and the vertical axis is the concordance of depth-specific, case-mix, and process evidence; position denotes qualitative direction, not measured distance. The classes are causal-interpretive categories rather than a numerical score; additional process or surveillance evidence can change the defensible classification. Class E (indeterminate) is shown as a separate off-map category because missing depth, process, or ascertainment data preclude placing a study on the plane at all. The four-step sequence described in Section 5.2 is the operational route into the matrix; it is rendered as a decision tree in Figure 5, which is the form intended for routine use by surveillance teams. The letters A–E denote the five SSI-SVF classes: A, probable true improvement; B, apparent improvement; C, masked deterioration; D, mixed deterioration; and E, indeterminate. The arrows indicate the direction of increasing or decreasing concordance and recorded SSI signal.

**Figure 5:**
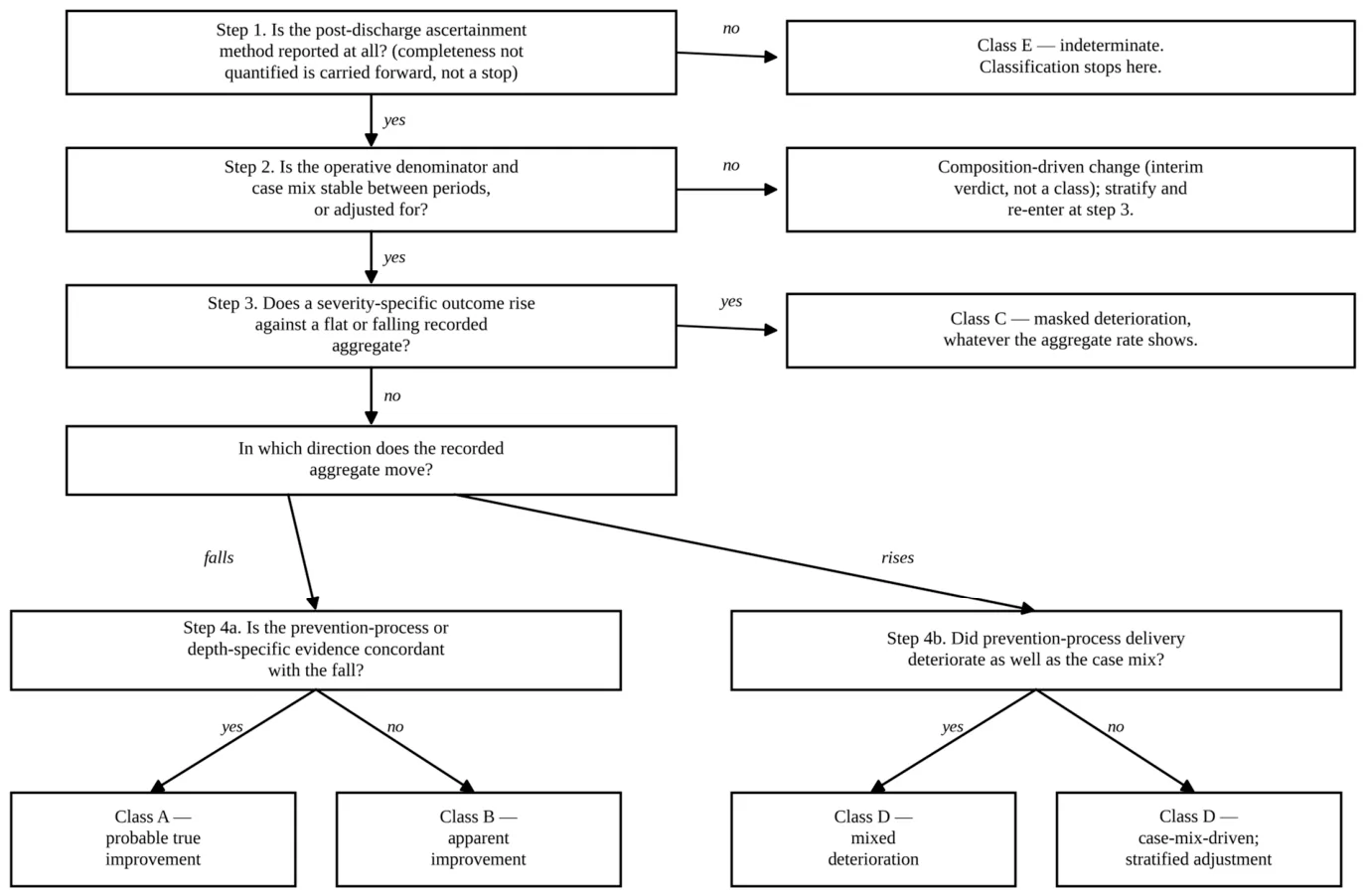
SSI-SVF decision tree. The tree renders the four-step sequence of Section 5.2 in the order in which a surveillance team would apply it and reaches the five classes that Figure 4 shows as a matrix. Class D is reached in its mixed and its case-mix-driven variant, as Table 2 also distinguishes them, and the step 2 exit is an interim verdict rather than a class. Ascertainment is read first because an unstable detection probability makes every later comparison uninterpretable.

Synthesis proceeded abductively. We first compared reported directions without imposing a framework, then examined studies that appeared inconsistent with the emerging explanation, and finally refined the framework around those deviant cases. No meta-analysis was performed, because procedure mix, urgency, depth definitions, follow-up, and calendar contrasts differed in ways that were not nuisance heterogeneity but the central explanatory variables. Quantitative results are therefore presented within studies, not pooled across studies. Because the synthesis is interpretive rather than statistical, we state the standard applied to it. A mechanism is described as supported only where a study reports the domain that would evidence it; as plausible where that domain is unmeasured but the reported direction is consistent with it; and as undetermined otherwise. Interpretive statements in Section 4, Section 5 and Section 6 follow it, the terms are used explicitly in Section 4.1, Section 4.2 and Section 4.3 where a classification turns on them, and no interpretation is drawn anywhere from a domain that a study did not report. Table 1, Table 2 and Table 3 were prepared directly in Microsoft Word. Figure 2 was plotted in GraphPad Prism v.9.3.0.3. (GraphPad Software, San Diego, CA, USA), and Figure 1, Figure 3, Figure 4 and Figure 5 are original schematic diagrams drawn by the authors; the values shown in Figure 2 are transcribed from the cited publications without smoothing, pooling, or re-estimation.

**Table 1:** Signal-validity audit of core comparative studies. Classes refer to Figure 4: A, probable true improvement; B, apparent improvement; C, masked deterioration; D, mixed deterioration; E, indeterminate. Mixed labels indicate that available information supports more than one interpretation.

Study/Setting	Direct SSI Signal	Ascertainment	Case-Mix, Depth and Process Information	Class
Ganam et al. [13], Israel; abdominal surgery; matched early-pandemic comparison	SSI 4.5% → 1.6% after 1:1 matching	CDC depth categories; routine office follow-up to 30 days plus emergency-room readmission monitoring	Urgency, approach and comorbidities matched; enhanced hygiene described; no quantitative bundle-compliance measure	A/B
Smith et al. [14], USA; multicenter NHSN procedures; colorectal subgroup	Non-significant decrease in matched colorectal procedures	NHSN definitions/window; publication does not quantify centre-period post-discharge completeness	Matching and standardized infection ratio; limited prevention-process data	B/E
Strobel et al. [19], Germany; elective IBD bowel resection	Overall SSI 31.8% → 25.8%; abscess 0.9% → 7.2%	CDC 30-day outcome; mailed questionnaire; 57.5% response; no routine day-30 examination	More contaminated/dirty wounds; depth reported; shorter stay; no bundle-fidelity measure	C
Vicentini et al. [15], Italy; 30-hospital abdominal surveillance network	5.65% baseline → 3.8% (2020) → 7.24% (2021)	ECDC-based prospective 30-day surveillance; visits/telephone; incomplete follow-up excluded	Urgency, approach, cancer indication and risk index measured; depth available; no process adherence	A/D over time
McLoughlin et al. [16], Canada; NSQIP, multiple surgical disciplines	Overall 4.5% → 2.8%; early general-surgery level change, then upward trend	NSQIP 30-day outcome architecture; post-discharge mode/completeness not separately reported	Interrupted time series and adjustment; no direct bundle-compliance data	A/B
Atumanyire et al. [20], Uganda; emergency laparotomy	16.7% → 17.6% (not significant)	Retrospective records; post-discharge completeness not clearly reported	Emergency-only pathway; depth and prevention-process reporting limited	E
Keske et al. [21], Türkiye; 17 hospitals, mixed procedures	0.79% → 0.87% → 0.46% → 0.50%	CDC-based surveillance; period-specific post-discharge completeness not reported	Large denominator and case-mix variables; limited procedure-specific/depth trend analysis	E
Alshahrani et al. [22], Saudi Arabia; abdominal surgery, 2019–2022	SSI 3.9% → 8.1%	CDC classification; minimum 30-day follow-up; inpatient daily wound review; outpatient method not fully detailed	Laparotomy/emergency/exploratory share ↑; prophylaxis use ↓; depth reported	D

Note: ↑ indicates an increase; ↓ indicates a decrease; ↔ indicates no material change/stability relative to the baseline period.

**Table 2:** Triangulation rules for interpreting recorded SSI changes. Arrows indicate direction relative to the relevant baseline.

Observed Pattern	Evidence That Must Be Read with It	Most Defensible Interpretation
Recorded SSI ↓; deep SSI ↓/↔	30-day ascertainment stable/complete; case mix adjusted; prevention adherence improved or remained high	Probable true improvement, while residual confounding remains
Superficial SSI ↓; deep outcomes not reported	Post-discharge contact reduced, response rate low, or discharge earlier	Apparent improvement; under-detection is a leading alternative
Overall SSI ↔/↓; organ/space SSI, abscess or reintervention ↑	Contaminated/emergency share ↑ and superficial follow-up incomplete	Masked deterioration; aggregate “any SSI” should not be the primary interpretation
SSI ↑; emergency/open/contaminated share ↑	Bundle adherence stable and surveillance stable	Case-mix-driven deterioration is plausible; stratified adjustment required
SSI ↑; case mix worsens and prophylaxis/process adherence ↓	Both pathway and process indicators change	Mixed system failure; do not attribute the increase to one mechanism
Post-pandemic SSI ↑ after a 2020 fall	Follow-up and volume recover; patient risk returns or exceeds baseline	Rebound may represent renormalized detection, case-mix recovery, true deterioration, or a combination

Note: ↑ indicates an increase; ↓ indicates a decrease; ↔ indicates no material change relative to baseline.

**Table 3:** Minimum reporting set for interpretable SSI comparisons during a health-system shock.

Domain	Required Variables	Why It Is Necessary
Denominator and pathway	Number of operations; procedure; indication; elective/urgent/emergency; open/minimally invasive; cancellations or backlog phase	Defines population burden and case-mix weights
Baseline risk	Age, ASA, comorbidity, frailty/nutrition where relevant, wound class, contamination, malignancy stage, physiologic severity	Separates selection from change in care
SSI outcome	Superficial, deep incisional and organ/space SSI; exact definition; 30/90-day window; date of onset	Prevents masking by an unstable composite
Ascertainment	Inpatient review, clinic, telephone/video, primary-care linkage, readmission capture; completion rate by period	Makes detection probability visible
Prevention process	Antibiotic choice/timing/redosing; skin preparation; normothermia; glycemia; bowel-preparation elements; wound protector and closure practice where relevant	Distinguishes bundle fidelity from general “hygiene”
Severe downstream outcomes	Abscess, imaging/drainage, reoperation, readmission, sepsis, mortality and competing events	Triangulates masked deterioration
Time structure	Monthly/quarterly series with policy and wave dates; pre-existing trend; recovery phase	Avoids treating a multi-phase shock as a binary exposure
Data provenance and reproducibility	Variable dictionary and coding system; missingness by period; label source and adjudicator; versioned extraction logic; code availability where applicable	Supports reproducibility and external validation and makes undocumented changes in detection probability visible

## 3. From a Recorded SSI Rate to a Valid Clinical Signal

### 3.1. Three Non-Equivalent Outcomes

Three outcomes are frequently conflated. First, population burden is the number of detected SSI events generated by a health system over a period. Second, recorded per-procedure risk is detected SSI divided by operations performed. Third, true per-procedure risk is the probability that an operated patient develops SSI, whether or not it is detected. During mass cancellation, population burden can fall simply because the number of operations falls, even if risk among the patients still operated rises. Conversely, restored follow-up can increase the recorded rate while true risk is unchanged.

This distinction matters operationally. A hospital may correctly report fewer SSI events during a shutdown, yet it would be incorrect to infer that surgery became safer. Similarly, a post-pandemic rebound can represent restored volume, restored detection, a less selected elective population, or genuine deterioration. Any review that uses “SSI decreased” without naming the estimand risks combining these different phenomena.

### 3.2. A Measurement-Aware Decomposition

Let k index clinically meaningful strata such as procedure × urgency × wound class × SSI depth. For calendar period t, the recorded per-procedure rate can be written conceptually as

R_recorded,t_ = Σ_k_ w_k,t_ × p_k,t_ × q_k,t_

where w_k,t_ is the proportion of operations in stratum k; p_k,t_ is the true probability of SSI in that stratum; and q_k,t_ is the conditional probability that an SSI is detected and recorded. Prevention and disease severity act mainly on p; cancellation and pathway reorganization act on w; follow-up, discharge, readmission capture, and diagnostic intensity act on q. This is the measurement core of SSI-SVF. The equation is an interpretive identity, not a fitted prediction model, and p is not presumed to be directly observed. Its purpose is to show why a calendar-period coefficient cannot, by itself, identify a change in biological risk. The expression also treats p and q as separable within a stratum, which is a simplification. Within the same stratum, a more severe infection is both more likely to arise under physiological stress and more likely to come to attention through readmission, imaging, or reintervention, so p and q may be positively correlated. The expression is therefore a conceptual heuristic for locating where a recorded change could have originated, not a statistical estimator to be fitted.

The formulation also clarifies aggregation bias. If low-risk elective procedures disappear, the aggregate w distribution shifts toward higher-risk surgery. If high-risk emergency cases are simultaneously delayed, p can rise. If superficial wound review then becomes less complete, q can fall. The final aggregate may increase, decrease, or remain unchanged depending on the relative size of these changes. The same recorded direction can therefore arise from different mechanisms, and opposite directions can arise from the same health-system shock. Figure 3 sets out the same decomposition schematically.

### 3.3. Depth-Dependent Ascertainment

Post-discharge surveillance materially increases SSI detection, and its contribution varies by procedure and infection type [26]. Superficial infections may be managed in primary care, by telephone, or without readmission; they are therefore especially vulnerable to changes in routine wound review. Deep and organ/space infections are not immune to under-ascertainment, but they are more likely to produce systemic illness, imaging, drainage, reoperation, or readmission. A fall in superficial SSI accompanied by stable or rising organ/space infection is therefore not merely a change in severity distribution; it is also a warning that aggregate surveillance sensitivity may have changed.

Depth-specific divergence is not unique to COVID-19. In 279,730 colorectal resections from 2013 to 2020, superficial and deep incisional SSI decreased while organ/space infection increased [27]. A subsequent NSQIP trend study found that elective colorectal SSI improved more than emergency SSI and that organ/space infection increased more in emergency surgery [28]. These longer-term data strengthen the argument that “any SSI” is an unstable composite when pathway and depth are not reported.

## 4. Direct Comparative Evidence Re-Read Through Signal Validity

### 4.1. Lower Recorded Rates: Possible Benefit, but Not a Single Mechanism

Ganam et al. compared abdominal operations before and during the early pandemic and used 1:1 propensity matching for age, sex, urgency, approach, and major comorbidities [13]. Follow-up included routine office visits for 30 days and monitoring of emergency-room readmissions. SSI fell from 4.5% to 1.6% in matched cohorts. Because the follow-up window and major measured case-mix variables were relatively well specified, this study provides stronger support for a real reduction than a simple in-hospital comparison. Nevertheless, it did not quantify period-specific adherence to each prevention-bundle component, so enhanced hygiene remains a plausible explanation rather than an isolated causal effect.

Smith et al. analyzed 29,904 NHSN procedures and matched COVID-era cases to historical controls [14]. The colorectal subgroup showed a non-significant decrease. The NHSN framework provides standardized outcome definitions, but the publication did not report centre- and period-specific completeness of post-discharge surveillance or prevention-process adherence. The result is therefore consistent with improved prevention, selection, or ascertainment change, but it does not discriminate among them. On the standard set out in Section 2.4 the prevention mechanism is therefore undetermined here rather than merely unconfirmed.

McLoughlin et al. used ACS NSQIP data from 2015 to 2021 and found lower overall SSI during the pandemic (2.8% vs. 4.5%) [16]. Their time-series analysis is more informative than a static before/after comparison: SSI was already trending downward before pandemic onset, the abrupt decrease was statistically evident only in general surgery, and the trajectory then turned upward during the pandemic. This is precisely the pattern that a single pandemic-period average would conceal. The study supports an early change in recorded SSI but weakens any claim of a durable, uniform “COVID bundle” effect.

### 4.2. Flat Overall SSI with Deeper Deterioration

Strobel et al. provide the clearest example of why depth and ascertainment must be read together [19]. In elective bowel resection for inflammatory bowel disease, overall SSI was not significantly different (31.8% before vs. 25.8% during the pandemic; adjusted OR 0.94), yet postoperative intra-abdominal abscess increased from 0.9% to 7.2%. The pandemic cohort also contained more contaminated/dirty wounds and had a shorter median hospital stay. Thirty-day surveillance relied on a mailed questionnaire with a 57.5% response rate and no routine clinical examination on day 30. The most defensible interpretation is not “SSI improved” or “SSI worsened” in isolation. It is a mixed signal: a more adverse operative substrate and more severe intra-abdominal infection coexisted with a recorded aggregate that was vulnerable to incomplete superficial-SSI capture.

In a low-resource emergency-laparotomy cohort from Uganda, Atumanyire et al. found no statistically significant difference in SSI (16.7% before vs. 17.6% during the pandemic) [20]. The result is clinically important because it shows that emergency surgery did not inevitably deteriorate in every setting. However, retrospective file review and limited description of post-discharge case finding constrain the conclusion to recorded SSI among observed patients. Stability may represent resilient prevention, insufficient power, or incomplete detection; the aggregate direction alone cannot choose among these explanations. The mechanism is accordingly undetermined.

### 4.3. Non-Monotonic and Higher Rates Reveal Boundary Conditions

The Italian SNICh network offers unusually strong surveillance information [15]. Thirty hospitals used an ECDC-based protocol with prospective 30-day follow-up, post-discharge visits or standardized telephone interviews, and exclusion of incomplete follow-up. Among 7605 abdominal procedures, the crude SSI rate was 5.65% in 2018–2019, 3.8% in 2020, and 7.24% in 2021. Adjusted analyses showed lower odds for non-cancer surgery in 2020 and higher odds in 2021. Because q was comparatively stable and documented, the reversal is more plausibly linked to changing w and p: the 2021 cohort had higher ASA and infection-risk scores, while volume and procedure composition were recovering. The study demonstrates that “the pandemic period” is not a single exposure and that early reduction and later rebound can both be valid within the same system.

Keske et al. reported another non-monotonic pattern across 17 Turkish hospitals: SSI rates rose slightly from 0.79% in 2019 to 0.87% in 2020 and then fell to 0.46% in 2021 and 0.50% in 2022 [21]. The study provides valuable multicenter incidence and antimicrobial-resistance data, but procedure-specific rates and post-discharge completeness were not the main analytic focus. Its contribution is therefore a system-level trend description rather than identification of a pathway-specific pandemic mechanism.

Alshahrani et al. are particularly informative because their cohort contradicts a simple assumption that pandemic hygiene should lower SSI [22]. Among 809 abdominal procedures with at least 30 days of follow-up, SSI increased from 3.9% to 8.1%. At the same time, laparotomy increased from 32.9% to 49.5%, emergency surgery from 11.6% to 17.3%, exploratory laparotomy from 8.6% to 25.8%, and antibiotic-prophylaxis use fell from 97.7% to 88.9%. The higher rate is therefore not a “discordant elective signal”; it is a mixed deterioration in w and p, with direct evidence of both a higher-risk case mix and weaker prevention-process delivery. Plummer et al. independently showed that urgent cases during pandemic disruption were less likely to receive several recommended preventive practices, including correct antibiotic selection/timing and chlorhexidine-alcohol preparation [23]. Together, these studies define a boundary condition: respiratory infection-control intensification cannot be assumed to preserve the surgical bundle when workflow resilience is poor. Because both domains are reported rather than inferred, the mechanism is supported in this cohort rather than merely plausible.

Figure 2 places the direct SSI signal from each core comparative study. The heterogeneity of direction is immediately visible, and Panel B isolates the mechanism behind Class C: an aggregate rate that looks flat or improved while a severity-specific outcome moves the opposite way.

### 4.4. What the Audit Adds

The audit changes the synthesis in three ways. First, it prevents studies with very different q values from being treated as if they measured the same outcome. Second, it shows that no core report simultaneously measured all information needed to isolate a biological pandemic effect: fixed and complete post-discharge surveillance, procedure × urgency × depth stratification, case-mix adjustment, and direct prevention-process fidelity. Third, it turns “conflicting results” into a set of identifiable missing variables. The problem is not only that the studies are heterogeneous; most were never set up to answer the causal question that is routinely asked of them.

Only one core abdominal cohort quantified a prevention-process indicator that changed across periods and could be paired directly with the outcome [22]. Several studies used a nominal 30-day window, but the method or completeness of post-discharge ascertainment was often incompletely reported. These omissions are not minor reporting defects. They determine whether a lower rate can be called prevention success, whether a flat rate can conceal deeper harm, and whether a higher rate reflects the operated population or the quality of care.

## 5. The SSI-SVF Signal-Validity Matrix

### 5.1. Five Interpretive Classes

The synthesis supports five classes of interpretation (Figure 4). They are deliberately non-numeric because the relevant evidence does not form a validated scale and because a single study can legitimately occupy more than one class.

- Probable true improvement: recorded SSI decreases while surveillance is stable and complete, case mix is stable or adjusted, and process or depth-specific evidence points in the same direction.
- Apparent improvement: overall or superficial SSI decreases, but follow-up weakens, the denominator changes materially, or deep outcomes are missing.
- Masked deterioration: the headline rate is flat or lower while organ/space infection, abscess, reintervention, contamination, or other severity indicators rise.
- Mixed deterioration: recorded SSI rises alongside a higher-risk operative mix and/or direct evidence that prevention-process adherence deteriorated.
- Indeterminate: the report provides an aggregate direction without enough information on urgency, depth, case mix, process, or ascertainment to infer mechanism.

Assignment follows a fixed reading of four of the seven audit domains in Section 2.3—post-discharge method and completeness, SSI depth, case mix, and prevention-process fidelity—rather than an overall impression; the remaining three domains qualify a reading but do not by themselves change the class. Each of the four is placed in one of three states: reported and favourable, reported and unfavourable, or not reported. Class A requires a falling recorded rate with all four reported and favourable, that is, post-discharge ascertainment described with its completeness quantified and stable between the periods compared, case mix measured or adjusted, and at least one depth-specific or prevention-process indicator moving concordantly with the aggregate. Class B applies when the rate falls but at least one of those domains is reported and unfavourable. Class C requires a flat or falling aggregate together with a reported rise in a severity-specific outcome, and takes precedence over A and B whenever such a rise is reported. Class D requires a rising aggregate together with a documented adverse shift in case mix, in prevention-process delivery, or in both; where only the case mix is shown to have worsened while process delivery and surveillance remain stable, the deterioration is case-mix-driven and stratified adjustment is required before any further reading. Class E applies when ascertainment, depth, and prevention-process information are together insufficient for any of the preceding conditions to be tested, however well the case mix is described. For prospective application we suggest treating a between-period difference in documented follow-up completeness of more than ten percentage points as not stable.

A dual label records the third state. Where a domain is not reported at all, rather than reported and unfavourable, the study is compatible both with the class that would follow if that domain were favourable and with the class that would follow if it were not; the label names both, and the unreported domains are the data that would resolve it. Ganam et al. and McLoughlin et al. are labelled A/B because the recorded rate falls, case mix is handled, and the analysis is structured enough for the fall itself to be interpretable, while two domains are left open in each: prevention-bundle adherence by period, and the completeness of post-discharge follow-up [13,16]. Had both been shown to be maintained, either study would satisfy Class A; had either been shown to have changed, both would fall to Class B. The second element of a dual label is not always the adjacent class. Where the unreported domains also remove the ground for reading the direction at all, the alternative is Class E rather than a lower class: Smith et al. is labelled B/E on that basis, because neither depth nor period-specific completeness is reported and the colorectal decrease was not statistically significant, so there is no interpretable signal left to downgrade [14]. Vicentini et al. is labelled A/D over time for a different reason again: the study reports two successive calendar contrasts within one prospectively surveyed network, with follow-up completeness documented and depth available, and the 2020 and 2021 contrasts satisfy the conditions of different classes [15]. Users of the framework should record the label together with the domains that produced it, so that a later report supplying them can reclassify the study without reopening the original reading.

### 5.2. Triangulation Rules

Table 2 is applied as a fixed four-step sequence, which is the form we recommend for surveillance teams. First, establish whether the post-discharge ascertainment method is reported at all; if it is not, classification stops at Class E, while a method that is described but whose completeness is not quantified is carried forward as an open domain rather than treated as a stop. Second, establish whether the operative denominator and case mix are stable or adjusted; if they are not, the aggregate change is attributed to composition until shown otherwise, and the study re-enters the sequence only after stratification. Third, read the depth-specific outcomes; if a severity-specific outcome rises against a flat or falling aggregate, classify as masked deterioration whatever the aggregate rate shows. Fourth, and only then, read the prevention-process evidence, which separates probable true improvement from apparent improvement when the rate falls, and mixed deterioration from case-mix-driven deterioration when it rises. If ascertainment completeness, depth, and prevention process are all still open at the end of the sequence, no condition can be tested and the study is Class E. Figure 4 summarizes the positions that result; the sequence above is the route to them, and Figure 5 renders the same sequence as a decision tree for use at the point of surveillance reporting.

### 5.3. Why “Resilience” Must Be Measured, Not Invoked

Health-system resilience is often used as a post hoc explanation for why one centre improved and another deteriorated. The present framework makes the term operational. Resilience is not a background label; it is the capacity to keep p-reducing processes and q-preserving surveillance functioning while w changes under stress. Measurable indicators include correct antimicrobial selection and timing, completion of skin preparation, glycemic control, availability of minimally invasive surgery and source control, protected emergency theatre access, follow-up completion, and time to presentation [29].

This operationalization explains why universal masking or environmental cleaning cannot be assumed to act as a surgical bundle. Respiratory precautions may coexist with missed prophylaxis, delayed source control, or reduced wound surveillance. Conversely, a centre can preserve stable emergency SSI despite severe system pressure if core surgical processes remain intact [20]. What explains the outcome is therefore local—how the pathway, the prevention processes, and the measurement system were arranged—rather than the pandemic period in itself.

## 6. Pathway and Procedure as Effect Modifiers

### 6.1. Elective Surgery: Denominator Compression and Selection

Global modelling estimated that 28.4 million operations would be cancelled or postponed during 12 weeks of peak disruption [6]. This contraction changes both the number of operations and the identity of the operated patient. Low-risk benign procedures may disappear, cancer surgery may be protected, and only patients judged sufficiently fit may proceed on COVID-free pathways [8]. The elective denominator is therefore “compressed” and selected. A lower per-procedure rate can reflect better prevention, but it can also reflect exclusion of patients who would ordinarily contribute infections.

The Italian network illustrates how selection is time-dependent: volume fell sharply in 2020, then partially recovered in 2021 while patient severity and SSI rose [15]. A robust elective analysis should therefore report both event burden and per-procedure risk, with the procedure distribution, indication, approach, and risk index for each period. Without these data, a lower rate is not transportable to the pre-pandemic or backlog-recovery population.

### 6.2. Emergency Surgery: Delay, Contamination, and Process Vulnerability

Emergency abdominal disease cannot be cancelled in the same way, but access can be delayed. German and Swiss cohorts documented more severe acute presentations, higher inflammatory markers, and more complicated appendicitis during pandemic restrictions [30,31]. A multicenter Italian study linked delayed access to increased emergency-surgery morbidity [32]. These findings do not prove that every emergency SSI increased, but they establish a credible upward pressure on p through perforation, contamination, and physiological compromise.

Emergency pathways are also more vulnerable to process failure. Urgency can reduce the time available for optimization and is associated with lower compliance with several prevention steps during disruption [23]. The 2025 colorectal trend study found a widening elective–emergency difference, particularly for organ/space infection, even outside a simple pandemic comparison [28]. This supports treating urgency as an effect modifier rather than merely another adjustment variable.

Emergency abdominal syndromes also show that the prognostic meaning of routinely measured variables can change when the care environment changes. In acute pancreatitis, a propensity-score-matched comparison found worse outcomes during the pandemic and especially among patients with concomitant SARS-CoV-2 infection [33], while a companion analysis reported period-dependent performance of conventional and newly proposed systemic inflammation indices [34]. In abdominal trauma, co-occurrence and multivariable analyses identified patterns of management and prognosis that were not captured by a simple before/during indicator [35]. These studies did not estimate SSI and are therefore mechanistic/contextual rather than direct SSI evidence. Their relevance to SSI-SVF is that p is not a fixed patient property: it can reflect disease severity, host inflammatory state, treatment access, and the transportability of prognostic relationships, all of which can change during a system shock.

### 6.3. Cholecystitis as a Natural Experiment in Pathway Resilience

Acute cholecystitis spans elective, urgent, and emergency pathways and therefore exposes the consequences of local policy choices. The CHOLECOVID study reported a shift toward greater disease severity and reduced cholecystectomy, with an indirect increase in 30-day mortality [36]. A systematic review documented broad adoption of conservative management and percutaneous drainage [37]. Some centres subsequently described difficult delayed operations, bile spillage, wound complications, and staffing-related harm [38,39], whereas others maintained early operative pathways without excess complications. This variation is not noise. It shows that whether the case-mix and prevention components push the rate up or down depends on how each centre reorganized its pathway.

### 6.4. Colorectal Surgery: Why Depth Cannot Be Collapsed

Colorectal surgery combines high baseline SSI risk with a large contribution from organ/space infection. Longer-term NSQIP analyses show declining superficial and deep incisional infection but rising organ/space infection [27], and a widening disadvantage for emergency compared with elective colorectal surgery [28]. These patterns mean that an aggregate colorectal SSI rate can remain stable even while the clinically more consequential component worsens. Pandemic studies should therefore report superficial, deep, and organ/space outcomes separately and should not use a fall in superficial SSI as evidence that source control or anastomotic outcomes improved.

Colorectal case mix may also differ in biological dimensions not captured by stage, urgency, or wound class. Serum and tissue analyses of substance P, calcitonin gene-related peptide, and their receptors have shown associations with disease stage and tumour differentiation in colorectal adenocarcinoma [40]. This evidence does not establish an SSI mechanism and should not be used to infer infection risk. It serves as an example of latent biological heterogeneity that may remain outside routinely adjusted w strata and should enter p only when prospectively linked to SSI outcomes.

## 7. Implications for Research, Surveillance, and Practice

### 7.1. A Minimum Reporting Set for Future System Shocks

The literature becomes substantially more informative if every period comparison reports the variables that determine w, p, and q. The proposed minimum set is designed for routine surveillance rather than a research-only dataset. It is given in Table 3.

### 7.2. Recommended Analytic Design

Future studies should prefer segmented time-series or controlled interrupted time-series designs over a single pre/post split when sufficient observations are available. The 2020 restriction phase, later pandemic adaptation, and backlog-recovery phase should be modelled separately. Analyses should report both the total number of SSI events and risk per operation, stratify by procedure, urgency, and depth, and adjust for wound class and other major risk factors. Time-to-event methods should retain the surveillance window and account for death as a competing event where relevant.

Process and outcome data should be linked. A claim that intensified hygiene reduced SSI is considerably stronger when correct prophylaxis, skin preparation, glycemic control, and follow-up completion are measured rather than assumed. Conversely, a rise in SSI can be more usefully targeted when the analysis shows whether the dominant change was more contamination, delayed presentation, loss of minimally invasive access, or missed bundle steps. This is where the framework earns its place: it turns a calendar-period association into a specific, answerable question about the surgical system.

### 7.3. Surveillance Policy

Post-discharge surveillance should be treated as core infrastructure, not an optional extension of inpatient infection control. Programmes should publish follow-up completeness by procedure and period, retain multiple contact modes, and link hospital records to readmission, emergency, and—where feasible—primary-care data. Remote review may improve access, but its diagnostic performance and escalation pathways should be validated. A surveillance system that changes sensitivity during a crisis cannot serve as a stable quality denominator unless that change is measured.

The same principle applies after the crisis. Restored clinic review can create an apparent post-pandemic “increase” by recovering previously missed superficial infections. Quality governance should anticipate this surveillance rebound rather than penalize services for improved detection. Electronic-record screening may supplement case finding, but heterogeneous postoperative-infection labels and limited validation mean that it should not replace standardized definitions or clinical adjudication [41].

## 8. Discussion

### 8.1. Main Synthesis

The direct evidence does not support a universal pandemic effect on abdominal SSI. More importantly, it cannot support one, because the observed rate is jointly generated by who underwent surgery, their true infection risk, and the probability of detection. Lower rates occurred in some early-pandemic and matched cohorts, stable rates occurred in some emergency settings, and higher or rebounding rates occurred when case mix and prevention processes changed. These are not mutually exclusive conclusions; they are different points in a three-component measurement system.

The clearest lesson from reading the studies alongside one another is how seldom the necessary variables were measured together. Studies commonly measured case mix without process fidelity, or used standardized definitions without reporting follow-up completeness, or presented any SSI without depth. Consequently, most calendar-period associations remain causally underdetermined even when statistically adjusted. The signal-validity matrix makes that underdetermination explicit rather than resolving it through narrative preference.

### 8.2. How This Framework Advances Beyond Existing Reviews

Previous reviews have appropriately described elective cancellation, delayed emergency presentation, enhanced infection control, and surveillance disruption. SSI-SVF advances the synthesis by linking these observations to a defined estimand and a set of falsifiable interpretive rules. It predicts, for example, that a genuine prevention benefit should persist when follow-up is complete and should be visible across compatible depth categories; that a flat aggregate with more organ/space infection and weaker superficial follow-up represents masked deterioration; and that a higher rate accompanied by both a riskier operative mix and lower prophylaxis adherence is mixed system failure rather than a simple pandemic effect.

The framework also reframes “discordant” studies. The Saudi cohort does not refute an elective–emergency distinction; it shows that an elective-dominant label is insufficient when the within-cohort share of laparotomy, emergency surgery, and exploratory surgery rises and prophylaxis delivery falls [22]. Similarly, the Romanian six-year SSI series contributes valuable microbiological and resistance information [42], but changes in length of stay among patients who already developed SSI cannot validate an ascertainment mechanism without the operative denominator and direct follow-up data. Keeping mechanism-compatible evidence separate from mechanism-confirming evidence matters, because conflating the two is how reviews end up overstating what they have shown.

### 8.3. Clinical Meaning

For clinicians, the matrix discourages false reassurance. A lower superficial-SSI rate during disrupted follow-up should not outweigh rising abscess, reintervention, or contaminated-case burden. For managers, it identifies process indicators that can be protected during stress. For surveillance teams, it treats follow-up completeness as an outcome in its own right. For researchers, it clarifies that the relevant comparison is not simply before versus during COVID-19, but the joint evolution of pathway composition, prevention delivery, and ascertainment.

## 9. Limitations

This is a critical narrative review, not a registered systematic review. Although the search was transparent and the direct evidence set was explicitly defined, iterative and language-restricted sampling can miss relevant reports. The framework and interpretive classes are author-generated and have not been externally validated; they should be tested by independent raters and in prospectively collected multicenter data. The audit judges what can be inferred from published reporting and is therefore vulnerable to information that may have been collected but not described. The assignment rules given in Section 5.1 make the classification reproducible in principle, but inter-rater agreement has not yet been measured and should be reported before the classes are used comparatively.

Most included evidence is retrospective and compares calendar periods that differ in many co-interventions. Even studies with strong surveillance cannot fully separate prevention from unmeasured selection. Some anchor studies include multiple surgical specialties, with abdominal or general-surgery findings extracted as subgroups. Policies, SARS-CoV-2 burden, vaccination, staffing, and surgical recovery differed by country and time, limiting direct transfer of a period label. The acute-pancreatitis, trauma, tumour-biology, and electronic-record surveillance sources are contextual and do not directly validate an SSI mechanism. Evidence on automated case finding remains heterogeneous and does not justify replacing clinical adjudication. Finally, the conceptual equation assumes that the major determinants can be represented through case-mix weights, true risk, and detection probability; interactions among them are expected, and the equation is not intended as a causal estimator.

## 10. Conclusions

Across the COVID-19 shock, abdominal SSI did not move in one universal direction. The more durable finding is interpretive: the recorded rate is a composite signal shaped by the operative denominator, the true probability of infection within each stratum, and the probability of detection. A lower rate is evidence of safer surgery only when case mix and ascertainment are stable or when concordant prevention-process and depth-specific outcomes support improvement. An unchanged aggregate can conceal a shift toward organ/space infection, while an increased rate can reflect simultaneous deterioration in case mix and prevention.

The Surgical-Site Infection Signal-Validity Framework (SSI-SVF) condenses this conclusion into a reusable rule: no calendar-period change in recorded SSI should be interpreted as a change in surgical safety until operative composition (w), true stratum-specific infection probability (p), and detection probability (q) have been examined together. This rule yields five defensible classes—probable true improvement, apparent improvement, masked deterioration, mixed deterioration, and indeterminate—and specifies a minimum reporting set: procedure and urgency, wound class, SSI depth and window, prevention-process fidelity, post-discharge follow-up completeness, severe downstream outcomes, and data provenance. The identity R_recorded,t_ = Σ_k_w_k,t_p_k,t_q_k,t_ is an interpretive identity, not a claim that p is directly observed or that the framework is a validated prediction model.

This has a direct policy consequence. The minimum reporting set cannot be delivered by individual departments acting alone, because the element that fails most often—completeness of post-discharge follow-up—is generated largely outside the operating unit. Interpretable comparison across a future system shock therefore requires a mandated national surveillance instrument rather than local goodwill: standardized definitions and windows applied uniformly; post-discharge case finding linked to readmission, emergency-department and, where feasible, primary-care records; follow-up completeness published by procedure and period alongside the rate itself; and participation tied to accreditation or to cost reimbursement. Without a reporting duty at that level, each successive shock will again produce rates that cannot be compared with the period preceding it.

SSI-SVF therefore shifts the unit of interpretation from a single percentage to a triangulated signal. Its value will be established not by further narrative agreement but by prospective multicenter data and reproducible application of the five classes by independent raters. Until then, the framework should be used as an explicit guard against false reassurance, masked deterioration, and causal overinterpretation of calendar-period SSI trends.

## Data Availability

No individual-level data were generated. The interpretability domains, classifications, and minimum reporting fields used in the narrative synthesis are reported in Table 1, Table 2 and Table 3.
